# Splendor in the Grass? A Pilot Study Assessing the Impact of Medical Marijuana on Executive Function

**DOI:** 10.3389/fphar.2016.00355

**Published:** 2016-10-13

**Authors:** Staci A. Gruber, Kelly A. Sagar, Mary K. Dahlgren, Megan T. Racine, Rosemary T. Smith, Scott E. Lukas

**Affiliations:** ^1^Cognitive and Clinical Neuroimaging Core, McLean Hospital Imaging CenterBelmont, MA, USA; ^2^Department of Psychiatry, Harvard Medical SchoolBoston, MA, USA; ^3^Department of Psychology, Tufts UniversityMedford, MA, USA; ^4^Behavioral Psychopharmacology Research Laboratory, McLean Hospital Imaging Center, McLean HospitalBelmont, MA, USA

**Keywords:** medical marijuana, cannabis, cognition, executive function, THC, cannabidiol

## Abstract

Currently, 25 states and Washington DC have enacted full medical marijuana (MMJ) programs while 18 states allow limited access to MMJ products. Limited access states permit low (or zero) tetrahydrocannabinol (THC) and high cannabidiol (CBD) products to treat specified conditions such as uncontrolled epilepsy. Although MMJ products are derived from the same plant species as recreational MJ, they are often selected for their unique cannabinoid constituents and ratios, not typically sought by recreational users, which may impact neurocognitive outcomes. To date, few studies have investigated the potential impact of MMJ use on cognitive performance, despite a well-documented association between recreational marijuana (MJ) use and executive dysfunction. The current study assessed the impact of 3 months of MMJ treatment on executive function, exploring whether MMJ patients would experience improvement in cognitive functioning, perhaps related to primary symptom alleviation. As part of a larger longitudinal study, 24 patients certified for MMJ use completed baseline executive function assessments and 11 of these so far have returned for their first follow-up visit 3 months after initiating treatment. Results suggest that in general, MMJ patients experienced some improvement on measures of executive functioning, including the Stroop Color Word Test and Trail Making Test, mostly reflected as increased speed in completing tasks without a loss of accuracy. On self-report questionnaires, patients also indicated moderate improvements in clinical state, including reduced sleep disturbance, decreased symptoms of depression, attenuated impulsivity, and positive changes in some aspects of quality of life. Additionally, patients reported a notable decrease in their use of conventional pharmaceutical agents from baseline, with opiate use declining more than 42%. While intriguing, these findings are preliminary and warrant further investigation at additional time points and in larger sample sizes. Given the likelihood of increased MMJ use across the country, it is imperative to determine the potential impact of short- and long-term treatment on cognitive performance as well as the efficacy of MMJ treatment itself.

## Introduction

Over the last several decades, although marijuana (MJ) users in the US have historically sought out MJ for recreational purposes, a growing number are exploring MJ for medical purposes. In fact, it is estimated that over 1.2 million medical MJ (MMJ) consumers are currently registered in the US (Procon.org^1^). According to Procon.org, although the majority of states have mandatory MMJ registration (CO, MA) other states have voluntary registration (e.g., CA, ME) or do not require registration (WA). While the number of current US MMJ consumers is only an estimate, it is likely that the number of certified patients will continue to grow as the public becomes increasingly aware of and open to the potential therapeutic effects of MMJ. Legal marijuana is considered the fastest growing market in the United States, with a current estimated value of $6.7 billion, which could reach 21.8 billion by 2020 (ArcView Market Research, 2016). In 1996, California became the first state to fully legalize MMJ and since then, another 24 states, and the District of Columbia have followed suit with full legalization for medical purposes, while an additional 18 states have limited MMJ laws, allowing only the use of products containing a specific non-psychoactive cannabinoid (cannabidiol [CBD]). Four states and the District of Columbia have also approved recreational MJ use, with several additional states pending legislation. Recent national surveys (Center for Behavioral Health Statistics and Quality, 2015; Johnston et al., 2015) report that MJ is retaining its status as the most widely used illicit drug for recreational purposes in the world; nearly 22.2 million Americans report use within the past month (Center for Behavioral Health Statistics and Quality, 2015). Further, while more than a million Americans are registered MMJ patients, this estimate does not include the unknown number of consumers currently taking hemp-derived products, marketed as high CBD-containing compounds (tinctures, oils, topicals), which are widely available from a number of vendors who do not require MMJ certification. Despite the rapid changes in policy, many legislators, consumers, physicians, and the general public remain misinformed about MJ. Although used for centuries as medicine by varied cultures across the world, in the US, MMJ became part of mainstream medicine in 1850, when it was added to the US Pharmacopeia. Physicians prescribed the use of MJ broadly for a range of indications including (but not limited to) pain, emesis, migraine, insomnia, epilepsy, and opium withdrawal (Birch, 1889; Potter, 1917; Grinspoon and Bakalar, 1997; Booth, 2003) and it remained widely available until 1937, when the marijuana tax law criminalized use of the substance. As anti-MJ sentiments grew across the country, it was removed from the pharmacopeia in 1942 and in 1970, the passage of the Controlled Substances Act (CSA) declared MJ a Schedule I substance and the cultivation, possession, and distribution of MJ became prohibited. According to the Drug Enforcement Administration (DEA), Schedule I drugs are those “with no currently accepted medical use, no demonstrated safety profile and a high potential for abuse…[they] are the most dangerous drugs of all the drug schedules with potentially severe psychological or physical dependence” (dea.gov^2^; Comprehensive Drug Abuse Prevention and Control Act of 1970^3^). This classification deems MJ more dangerous than other substances including cocaine, methamphetamine, and opiate-based drugs, which ironically are responsible for approximately 30,000 deaths per year (Centers for Disease Control and Prevention, 2015). In fact, opioid overdoses are now considered a national epidemic; the rate of opioid overdose deaths, including those related to both prescription pain relievers and heroin, has nearly quadrupled since 1999 (Centers for Disease Control and Prevention, 2015). Given its Schedule I classification, research studies exploring both potential risks and benefits of MMJ have faced numerous obstacles, forcing policy to outpace science in recent years. As the national climate warms toward MJ, research is slowly pushing forward. However, much is left to be explored before the gap between science and policy can begin to close.

A growing body of evidence suggests that recreational MJ use adversely impacts the brain, particularly during critical periods of neurodevelopment, including adolescence (For review: Crean et al., 2011; Jacobus and Tapert, 2014; Lisdahl et al., 2014). Numerous studies have shown that MJ users, particularly those who initiate use during adolescence, exhibit deficits across multiple cognitive domains. For example, MJ users who initiate use during adolescence exhibit deficits in attention (Ehrenreich et al., 1999; Cousijn et al., 2013; Becker et al., 2014) and processing speed (Fried et al., 2005; Medina et al., 2007; Lisdahl and Price, 2012; Jacobus et al., 2015). Furthermore, lower scores on measures of IQ (Pope et al., 2003; Meier et al., 2012; Crane et al., 2015) have been observed among adolescent MJ users, although recent work has questioned this finding (Jackson et al., 2016; Mokrysz et al., 2016), and a number of studies have reported poorer verbal memory among adolescent and adult MJ smokers (Tait et al., 2011; Auer et al., 2016; Shuster et al., 2016). Data also suggest that adolescent MJ use is strongly associated with poorer executive functioning (Fontes et al., 2011; Solowij et al., 2012; Crane et al., 2013; Dougherty et al., 2013; Tamm et al., 2013; Becker et al., 2014; Hanson et al., 2014; Winward et al., 2014; Jacobus et al., 2015; Sagar et al., 2015) even when deficits in other domains are not observed (Gruber et al., 2012a).

In contrast, although research is in its infancy, given what is currently known about MJ, it is possible that MMJ use may *not* lead to the same neurocognitive consequences that have been observed in recreational users. Although recreational and medical MJ are derived from the same plant species, there are inherent differences that exist between the two. As recreational users most frequently seek a mood altering, often “euphoric” or “mellow” state, they primarily utilize products with considerable amounts of THC, the main psychoactive ingredient in MJ (Wachtel et al., 2002; Zeiger et al., 2010). Over the last two decades the potency of recreational marijuana has significantly increased from approximately 4 to 12% between 1995 and 2014 in response to consumer demand (ElSohly et al., 2016). In contrast, MMJ users primarily initiate MMJ use as a means of symptom alleviation (Nunberg et al., 2013), and as such are likely to seek products for their therapeutic potential rather than to experience the psychoactive effects. They may therefore use products differently and purchase products with a markedly different chemical composition from more common recreational products. These MMJ products are often (but not always) high in other cannabinoids, such as cannabidiol (CBD) which has been touted for its therapeutic potential, and which is *not* psychoactive. CBD has become best known in recent years for its potential to treat those with intractable seizure disorders, specifically children with Dravet Syndrome or Lennox Gastaux Syndrome, and preliminary data from both anecdotal reports and recent clinical trials are promising. In a recent open-label trial in patients aged 1–30 with severe, intractable, childhood-onset, treatment-resistant epilepsy, Devinsky et al. (2016) reported that the median monthly frequency of motor seizures decreased from 30 per month at baseline to 15.8 per month during the treatment period in patients treated with Epidolex, a 98% purified CBD compound created by GW Pharma. CBD has also demonstrated promise in treating other conditions including chronic pain, multiple sclerosis (Giacoppo et al., 2015), and Huntington's disease (Consroe et al., 1991) as well as psychiatric and behavioral health conditions including anxiety (for review: Blessing et al., 2015) and psychosis (Zuardi et al., 2009; Leweke et al., 2012). Interestingly, some work suggests that CBD may have a pharmacological profile similar to that of antipsychotic medications (Zuardi et al., 2012). In addition to CBD, a host of other cannabinoids, many of which are non-psychoactive, are also often present in MMJ products, and becoming increasingly popular. Other phytocannabinoids, including cannabigerol (CBG), cannabinol (CBN), cannabichromene (CBC), tetrahydrocannabinolic acid (THCA), and tetrahydrocannabidivarin (THCV), have shown therapeutic potential and may also reduce some of the undesirable effects associated with THC. For example, cannabichromene (CBC), another abundant cannabinoid, has anti-inflammatory effects (Izzo et al., 2012) and has recently been shown to increase the viability of adult neural stem progenitor cells (NSPCs), essential for brain plasticity and suggestive of neurogenesis (Shinjyo and Di Marzo, 2013). In addition, cannabigerol (CBG) inhibits GABA uptake, has anti-inflammatory properties, and has also been touted as being neurogenic (Borelli et al., 2013; Valdeolivas et al., 2015), while tetrahydrocannabidivarin (THCV) has been shown to inhibit some of the negative cognitive and physiologic effects of THC and may be neuroprotective (Englund et al., 2016).

Despite the majority of states with MMJ laws and more than a million registered patients, no studies to date have utilized a pre- vs. post-design model to examine the specific impact of MMJ on cognitive performance as a primary outcome variable. As noted above, cognitive deficits are demonstrated in chronic, heavy, recreational MJ users who begin MJ use during adolescence (for review: Crean et al., 2011; Jacobus and Tapert, 2014; Lisdahl et al., 2014), and while some clinical trials of MMJ (particularly CBD) have been initiated in children for treatment-resistant epilepsy (Devinsky et al., 2016), the majority of those utilizing MMJ products are adults, and beyond the most critical period of neurodevelopmental vulnerability. In addition, it is likely that if physical or psychological symptoms are addressed by MMJ use, cognitive function may improve. For example, studies have reported that anxiety often interferes with both attention and executive function (e.g., Vytal et al., 2013); if MMJ products act as an anxiolytic for at least some patients as reported, this may result in better concentration and enhanced cognitive performance. Chronic pain has also been noted to impair cognitive performance, notably tasks requiring attentional and executive function (for review see Moriarty et al., 2011). Accordingly, if patients experience a reduction in pain-related symptoms as a result of MMJ treatment, it is likely that cognitive performance will improve relative to a pre-treatment assessment.

In order to evaluate the impact of MMJ use on cognitive function and determine the efficacy of MMJ in a broad sample of MMJ patients, we designed a longitudinal study which assesses MMJ patients at baseline and after 3, 6, and 12 months of MMJ treatment. Importantly, baseline measurements were taken prior to the initiation of MMJ treatment in order to obtain an “MJ naïve” assessment. Given the differences between MMJ and recreational MJ use and the reported potential for symptom alleviation in MMJ users, we hypothesized that MMJ patients would demonstrate improved cognitive performance on tasks of executive functioning, as well as improved clinical state and quality of life following MMJ treatment. This study is currently ongoing, and in this paper, we report our preliminary cognitive findings in addition to information regarding general health and clinical state measures as well as medication use, after 3 months of MMJ treatment.

## Materials and methods

### Participants

To date, 32 participants completed screening procedures, of which 24 MMJ users were successfully enrolled in the current study. Data from 11 patients' baseline and 3-month check-in visits (Visit 2) were available for preliminary analysis. In order to qualify for study entry, participants are required to either be MJ naïve or if they have a history of MJ use, they must report MJ abstinence for 10 or more years in an effort to ensure that recent MJ exposure does not impact results. Self-report MJ status was confirmed with urinalysis. Participants are also required to have a valid certification for MMJ, and may report seeking MMJ treatment for a variety of indications; the current sample (*n* = 11) reported MMJ certification for anxiety (*n* = 5), depression (*n* = 3), chronic pain (*n* = 7), sleep (*n* = 5), and other conditions (*n* = 6). Notably, 9 of 11 participants reported seeking out MMJ treatment for two or more conditions or symptoms.

### Study design

Prior to participation, study procedures were thoroughly explained, and all participants were required to read and sign an informed consent form. This document describes the procedures, risks, benefits, and voluntary nature of the study. All study procedures were approved by the Partners Institutional Review Board (IRB).

Prior to initiating MMJ treatment, all enrolled participants completed a neurocognitive battery as well as measures of clinical state, quality of life, sleep, and general health assessments. In addition, eligible patients completed neuroimaging sessions (i.e., functional and structural magnetic resonance imaging), which will be reported in subsequent publications. Following 3 months of regular MMJ treatment, participants returned for a check-in visit (Visit 2) and repeated all study measures. In addition, once patients began a regular MMJ use regimen, they were contacted by phone to complete monthly check-in visits, which assessed type, frequency and magnitude of MMJ use using a modified timeline follow-back procedure (TLFB; Sobell et al., 1998) and other queries. During check-in calls, patients were asked to provide qualitative information regarding product type and strain of MMJ products used as well as mode of use (i.e., joint, vaporizer, tincture, edibles, etc.) and quantitative information regarding frequency (episodes of MMJ use/week) and magnitude (doses/week) of use. These data were reviewed, clarified, and corroborated in person during Visit 2. Each participant also provided a sample of his/her most frequently used MMJ product, which was analyzed by an outside laboratory (ProVerde Laboratories, Inc.) in order to obtain cannabinoid constituent profiling for each product, providing information on THC and CBD levels as well as a number of other cannabinoids. These data will be examined in future investigations in order to determine the impact of constituent ratios on cognitive and clinical outcomes.

#### Cognitive and clinical assessments

All subjects completed the Wechsler Abbreviated Scale of Intelligence (WASI; Wechsler, 1999), which provides an estimate of overall cognitive functioning, to ensure an estimated IQ of 75 or higher. As a part of a larger neurocognitive battery, each individual completed several measures of executive functioning, including the Stroop Color Word Test, the Trail Making Test, the Wisconsin Card Sorting Test (WCST), and Letter-Number Sequencing subtest of the Wechsler Adult Intelligence Scale (WAIS). The Stroop Color Word Test (MacLeod, 1991) assesses the ability to inhibit an automatic, overlearned response. While the first condition of the task (Color Naming) assesses rapid naming abilities, the second condition (Word Reading) requires that the individual read words printed in black ink which also serves as a primer for the third task (Interference) where they must inhibit the natural tendency to read the words aloud and instead must name the color of the ink words are printed in. During the Interference condition, each word is printed in a color incongruous to the actual word (i.e., *red* printed in green, or *blue* printed in red). The Trail Making Test (Lezak et al., 2004) is comprised of two timed conditions where participants must connect a series of dots. Trails A measures psychomotor function, visual scanning, and attention by asking participants to connected dots in numerical order. Trails B incorporates alternating set demands, requiring participants to alternate between numbers and letters (e.g., 1, A, 2, B, etc.), in order to measure cognitive flexibility and executive function. The WCST (Berg, 1948; Lezak et al., 2004) is a robust measure of executive functioning, which also assesses cognitive flexibility and set-shifting. All participants completed a computerized administration of the task in which they must match cards based on sorting rules and adjust to changing sorting rules based solely on feedback as to whether each match is either correct or incorrect. Finally, during the Letter-Number Sequencing task (Wechsler, 1997), participants are read increasingly longer strings of numbers and letters and are asked to repeat them, first citing the numbers in order, and then the letters in alphabetical order. Performance on this task is correlated with executive function and working memory abilities. Subjects completed all tasks at baseline and at the 3-month follow-up visit, with the exception of the WASI, which was administered only at baseline in order to obtain an IQ estimate.

In order to determine whether patients experienced any change in clinical symptoms or health-related measures which could potentially impact cognitive performance, each participant completed a battery of clinical state assessments, including the Profile of Mood States (POMS), Beck Depression Inventory (BDI), Beck Anxiety Inventory (BAI), and Barratt Impulsiveness Scale (BIS) as well as quality of life and general health questionnaires including the Pittsburgh Sleep Quality Index (PSQI) and Short-Form 36 Health Survey (SF-36). The POMS (Pollock et al., 1979) measures self-perceived mood, and generates subscores for feelings of vigor, confusion, tension, anger, depression, and fatigue, which combine to generate an overall measure of Total Mood Disturbance (TMD). The BDI (Beck et al., 1961) and BAI (Beck and Steer, 1990) are brief self-report measures that directly assess symptoms of depression and anxiety. The BIS (Patton et al., 1995) assesses self-reported impulsivity across three discrete domains (Attention, Motor, and Non-Planning), which together provide an overall composite score. The PSQI (Buysse et al., 1989) provides a measure of sleep quality over the past month. The SF-36 (Ware and Sherbourne, 1992) questionnaire is a multi-purpose, short-form health survey that yields an eight-scale profile of functional health and well-being scores for the following domains: physical functioning, physical role limitations, emotional role limitations, energy/fatigue, emotional well-being, social functioning, pain, and general health.

In addition, patients' use of conventional pharmaceutical medications was also tracked in order to determine whether MMJ use might be related to changes in other medication use. At baseline and during Visit 2, participants provided a list of medications used on at least a weekly basis. These medications were then coded into different classes, including opiates, antidepressants, mood stabilizers, benzodiazepines, sedatives, and muscle relaxants. Percent change data based on number of doses taken per week was calculated for medication use from baseline to Visit 2.

### Statistical analyses

Descriptive statistics (e.g., mean and standard deviation) were calculated for demographic and MMJ use variables. Paired *t*-tests were used to assess within-subjects changes from baseline to Visit 2. Given our hypotheses that MMJ treatment would be associated with improved cognitive performance, clinical state, and general health ratings, one-tailed *t*-tests were utilized throughout.

## Results

### Demographics

As reported in Table 1, participants (6 males, 5 females) enrolled in current study were between the ages of 32 and 74, with an average age of 48.91. Generally, participants were well-educated, having all earned at least a high school diploma, and were of at least average intelligence, as noted on the WASI. All reported using MMJ at least weekly since the initiation of regular MMJ treatment (1–7 days per week; 4.95 days on average). Half of the sample reported typical use of MMJ products more than once per day; patients reported MMJ use 1.78 times per day on average, resulting in an average weekly total of 9.30 episodes per week. With regard to product type and mode of use, the majority of participants (8 of 11) reported using flower products in some form (smoked or vaporized). Reported use of concentrates and oils (3 of 11) and “medibles” (MMJ edible products; 3 of 11) by participants was less frequent. Other modes of administration (i.e., topicals) were not reported among the current sample.

**Table 1:** **Demographics and MMJ use**.

**Demographic variable (*n* = 11)**	**Mean (SD)**
Age	48.91 (15.13)
Education (years)	15.82 (1.47)
WASI full scale IQ^a^	117.09 (7.49)
**MMJ use^b^**	
Days of MMJ use/week	4.95 (2.26)
Times/day used	1.78 (1.19)
Total MMJ use episodes/week	9.30 (8.86)
**Mode of use^c^**	**Number of participants**
Smoke (flower product)	5
Vaporize (flower product)	6
Vaporize (oil/concentrates)	2
Oil/concentrates (non-smoked/vaporized)	1
Tincture	3
Medibles^d^	3

a *WASI, Wechsler Abbreviated Scale of Intelligence*.

b *MMJ use reflects average use from the start of regular treatment through Visit*.

c *Based on 11 participants' reported mode(s) of use*.

d *Medibles, MMJ edible products*.

### Cognitive performance

As shown in Table 2, following 3 months of MMJ treatment, participants completed Trails A significantly faster (*p* = 0.02) and also tended to complete Trails B (*p* = 0.06) faster relative to baseline. Further, this improvement was not at the expense of task accuracy, as no difference in number of errors was observed across visits for Trails A or Trails B. On the Stroop Color Word Test, participants also demonstrated faster completion times and some improvements in task accuracy following 3 months of treatment. As compared to baseline, performance on the Color Naming condition was significantly faster (*p* = 0.01), again with no decrement noted in percent accuracy. Performance was virtually unchanged across visits on the Word Reading subtest, which is primarily utilized to prime participants for the Interference condition. During the Interference condition, participants also demonstrated faster completion times relative to baseline (*p* = 0.01). Although no improvement was detected for accuracy on the Interference condition, participants maintained their high levels of performance from baseline to their 3-month check in visits. Overall on the WCST, no changes in performance were observed for categories completed, correct responses or perseverative errors, and although some improvements were noted from baseline to Visit 2, these did not reach statistical significance. On the Letter Number Sequencing task, while the total number correct increased slightly from baseline to Visit 2, this difference did not reach statistical significance.

**Table 2:** **Cognitive performance at baseline (Visit 1) and after 3 months (Visit 2) of MMJ treatment**.

**Neurocognitive measure**	**Visit 1 Mean (SD)**	**Visit 2 Mean (SD)**	**1-tailed *t*-test *t* (*p*)**
**TRAIL MAKING TEST**			
Trails A time (sec)	**26.91 (6.63)**	**22.91 (3.89)**	**2.29 (0.02)**
Trails A errors	0.36 (0.51)	0.55 (0.82)	0.69 (0.25)
Trails B time (sec)	*64.18 (13.42)*	*54.00 (11.87)*	*1.72 (0.06)*
Trails B errors	0.18 (0.41)	0.18 (0.41)	0.00 (0.50)
**STROOP COLOR WORD TEST**			
Color naming percent accuracy	97.46 (1.81)	97.81 (1.83)	1.31 (0.11)
Color naming time (sec)	**59.55 (15.85)**	**56.91 (14.19)**	**2.84 (0.01)**
Word reading percent accuracy	99.55 (1.04)	99.27 (1.27)	0.67 (0.26)
Word reading time (sec)	44.09 (12.79)	43.46 (14.90)	0.69 (0.25)
Interference percent accuracy	*97.91 (1.81)*	*97.18 (3.06)*	*1.35 (0.10)*
Interference time (sec)	**106.00 (27.15)**	**96.27 (20.66)**	**2.77 (0.01)**
**WISCONSIN CARD SORTING TEST**			
Total categories completed	3.00 (1.10)	2.81 (1.16)	0.56 (0.29)
Total correct responses	45.00 (8.10)	46.36 (8.24)	0.51 (0.31)
Total concept level responses	39.18 (10.41)	41.91 (11.45)	0.84 (0.21)
Total perseverative errors	8.36 (4.65)	7.82 (5.12)	0.35 (0.37)
**LETTER-NUMBER SEQUENCING**			
Total correct	11.55 (2.54)	12.00 (2.83)	1.17 (0.14)

*Values in bold represent statistically significant differences from Baseline to Visit 2 (p ≤ 0.05), and italicized values represent statistical trends (p ≤ 0.10)*.

### Clinical ratings and pharmaceutical medication usage

As noted in Table 3, several discrete measures of clinical state and general health improved relative to baseline. Participants reported significantly lower levels of depression on the BDI (18.18 vs. 13.64, *p* = 0.04) as well as a trend for lower level of sleep disturbance (reflecting improved sleep) on the PSQI (9.63 vs. 7.25, *p* = 0.06). In addition, participants reported a moderate degree of decreased impulsivity on the BIS (Motor subscore: 22.91 vs. 21.46, *p* = 0.03). On the SF-36, participants also generally reported improved quality of life, including significant improvements on a scale measuring Energy/Fatigue (35.45 vs. 45.91, *p* = 0.02) as well a trend for fewer role limitations due to physical health (43.18 vs. 56.82, *p* = 0.07), which reflects how often patients “cut down the amount of time [they] spent on work or other activities,” “accomplished less than [they] would like,” “were limited in the kind of work or other activities,” or “had difficulty performing the work or other activities” as a result of physical health.

**Table 3:** **Mood and health ratings at baseline (Visit 1) and after 3 months (Visit 2) of MMJ treatment**.

**Rating scale**	**Visit 1 Mean (SD)**	**Visit 2 Mean (SD)**	**1-tailed *t*-test *t* (*p*)**
**CLINICAL RATING SCALES**			
***Profile of Mood States (POMS)***			
Vigor	15.64 (6.85)	15.46 (6.67)	0.11 (0.46)
Anger	10.00 (12.16)	9.73 (9.73)	0.09 (0.46)
Confusion	9.18 (6.90)	8.10 (4.70)	0.87 (0.20)
Tension	15.55 (11.24)	15.10 (10.55)	0.17 (0.44)
Fatigue	11.27 (8.49)	11.18 (9.08)	0.08 (0.47)
Depression	16.18 (19.04)	19.18 (18.48)	0.76 (0.23)
TMD	46.55 (57.18)	47.82 (52.73)	0.12 (0.46)
***Beck Depression Inventory (BDI)***			
Total	**18.18 (13.00)**	**13.64 (13.75)**	**1.97 (0.04)**
***Beck Anxiety Inventory (BAI)***			
Total	13.46 (12.74)	13.36 (12.18)	0.03 (0.49)
**IMPULSIVITY**			
***Barratt Impulsiveness Scale (BIS-11)***			
Attention	18.73 (4.56)	18.82 (4.36)	0.10 (0.46)
Motor	**22.91 (3.86)**	**21.46 (4.41)**	**2.10 (0.03)**
Non-planning	23.82 (6.57)	24.45 (5.66)	0.53 (0.30)
Total	65.46 (14.07)	64.73 (12.67)	0.30 (0.39)
**HEALTH AND QUALITY OF LIFE RATINGS**			
***Pittsburgh Sleep Quality Index (PSQI)***			
Total	*9.63 (4.84)*	*7.25 (3.69)*	*1.80 (0.06)*
***Short-Form 36 Healthy Survey (SF-36)***			
Physical functioning	68.18 (26.20)	65.91 (30.23)	0.23 (0.41)
Role limitations (physical)	*43.18 (43.43)*	*56.82 (43.43)*	*1.40 (0.10)*
Role limitations (emotional)	57.58 (44.95)	54.55 (47.78)	0.22 (0.42)
Energy/fatigue	**35.45 (25.34)**	**45.91 (24.47)**	**2.50 (0.02)**
Emotional well being	61.46 (27.56)	58.55 (27.20)	0.84 (0.21)
Social functioning	54.55 (29.72)	63.64 (31.35)	1.06 (0.16)
Pain	47.05 (34.15)	51.82 (30.95)	0.95 (0.18)
General health	54.55 (21.96)	55.46 (20.55)	0.20 (0.42)

*Clinical rating scales (POMS, BDI, BAI), lower scores reflect lower levels of clinical symptoms*.

*BIS-11, lower scores indicate lower levels of self-reported impulsivity*.

*PSQI, lower scores reflect improved sleep quality*.

*SF-36, higher scores indicate higher quality of life*.

*Values in bold represent statistically significant differences from Baseline to Visit 2 (p ≤ 0.05), and italicized values represent statistical trends (p ≤ 0.10)*.

Following 3 months of MMJ treatment, participants also reported reductions in the use of conventional pharmaceutical products. As shown in Table 4, percentage change [(Visit 2-Visit 1)/Visit 1] data revealed a notable decrease in weekly use across all medication classes, including reductions in use of opiates (−42.88%), antidepressants (−17.64%), mood stabilizers (−33.33%), and benzodiazepines (−38.89%). Interestingly, *t*-tests indicated trends for significant reductions in opiate (5.85 vs. 2.27, *p* = 0.08) and antidepressant (8.54 vs. 7.00, *p* = 0.09) use. Although sedative (−100.00%) and muscle relaxant (−100.00%) usage were notably decreased, these data were only based on one participant for each of these medication classes.

**Table 4:** **Weekly medication usage (doses/week) between baseline (Visit 1) and after 3 months (Visit 2) of MMJ treatment**.

**Medication class**	**Number of patients using medication**	**Visit 1 Mean (SD)**	**Visit 2 Mean (SD)**	**1-tailed *t*-test *t* (*p*)**	**Percent change Mean (SD)**
Opiates	6	5.85 (5.95)	2.27 (3.72)	1.66 (0.08)	−42.88% (51.90)
Antidepressants	7	8.54 (3.95)	7.00 (4.04)	1.49 (0.09)	−17.64% (37.05)
Mood Stabilizers	3	5.83 (2.02)	4.67 (4.04)	1.00 (0.21)	−33.33% (57.73)
Benzodiazepines	6	5.90 (10.44)	0.50 (1.19)	1.12 (0.16)	−38.89% (71.23)
Sedatives	1	5.00 (NA)	0.00 (NA)	–	−100.00% (NA)
Muscle Relaxants	1	3.50 (NA)	0.00 (NA)	–	−100.00% (NA)

## Discussion

Despite rapid changes in the law, many policy makers, consumers, physicians, and the general public remain misinformed about MJ. While a body of evidence has demonstrated alterations in brain structure and function secondary to recreational MJ use, particularly related to use during vulnerable developmental periods such as adolescence (For review: Crean et al., 2011; Jacobus and Tapert, 2014; Lisdahl et al., 2014), critical questions regarding the impact of MMJ use remain unanswered. To our knowledge, the current pilot study marks the first investigation to specifically examine cognitive performance and related measures in MMJ patients *prior to initiation of treatment* relative to performance following regular MMJ use. Preliminary results from this first phase of the study suggest that after 3 months of MMJ treatment, participants experienced some degree of improvement on tasks of executive functioning. In addition, no significant decrements in performance were noted across any measure of executive function completed by participants.

Further, results from the current study suggested some improvement in self-reported measures of clinical state and general health. MMJ participants reported significant improvement on measures of depression and impulsivity. Participants' ratings also indicated some improvement in sleep quality, which is likely related to ratings of significantly increased energy and decreased fatigue on self-report measures assessing quality of life. Study findings provide further evidence of improvements in medical and clinical symptoms secondary to MMJ use, consistent with other recent reports that have also demonstrated varying degrees of efficacy of cannabinoid-based therapies (Boychuk et al., 2015; Deshpande et al., 2015; Press et al., 2015; Whiting et al., 2015; Devinsky et al., 2016; Haroutounian et al., 2016; Wilkie et al., 2016).

In a survey study of California residents, Ryan-Ibarra et al. (2015) recently found that of the 5% of California who reported having ever used MMJ, 92% report that MMJ helped treat a serious medical condition, with pain being the most commonly reported indication for use. Notably, improvements observed in the current study occurred concomitantly with a reduction in reported use of pharmaceutical products (particularly opiates and antidepressants), which are used to treat many symptoms for which patients in this study initiated MMJ use. In a recent European study designed to assess the impact of MMJ on chronic pain, Haroutounian et al. (2016) also reported reductions in conventional pharmaceutical use among study patients, specifically with regard to opioid use. Similarly, a study examining physician records of 1655 patients seeking a physician certification at multiple MMJ evaluation clinics in California found that half of applicants reported using MJ as a substitute for a prescription medication (Nunberg et al., 2013). Given the current opioid crisis, it is critical to examine the potential role for cannabinoids, as many patients do not get full symptom relief from conventional opiate-based therapies and often complain about the myriad of associated side effects (Ballantyne and Shin, 2008; Rosenblum et al., 2008). In addition, it is important to consider a recent study which found that MJ users reported greater pain relief when MJ was used in combination with pharmaceutical opioids than when opioids were used alone (Degenhardt et al., 2015).

Several hypotheses may be considered for the observed improvements in the current pilot investigation. First, as postulated, participants experienced some amelioration of clinical symptoms. This reduction of symptomatology, in combination with reported improvements on other measures (improved sleep, less impulsivity), may result in the observed improvements in cognitive functioning. As previously noted, symptoms commonly reported in MMJ patients including anxiety and pain, have been associated with reduced cognitive performance (Moriarty et al., 2011; Vytal et al., 2013). Symptom improvement may therefore result in improved cognitive performance. Interestingly, two previous studies have noted a positive association between a history of MJ use and *improved* cognitive performance on measures of psychomotor speed, attention, working memory, executive functioning, and verbal learning in patients with bipolar disorder compared to patients without a history of marijuana use (Ringen et al., 2010; Braga et al., 2012). Further investigation is warranted, given the number of patients seeking MMJ treatment for symptoms known to interfere with cognitive function.

It is also possible that MMJ products themselves protect against the executive function deficits that have been widely reported in recreational MJ users (for review see Crean et al., 2011). Although results from the current study appear to be in stark contrast to those from some recreational MJ studies, the answer may lie in the inherent differences between MMJ and recreational MJ products and the differences between the two consumer groups. While THC potency is rising and CBD levels have decreased to barely perceptible levels in recreational MJ strains (ElSohly et al., 2016), some MMJ products contain higher amounts of CBD and other cannabinoids which may mitigate the adverse effects of THC on cognitive performance. Although research is limited in this area, Englund et al. (2013) found that administering CBD prior to intravenous administration of THC in healthy control participants resulted in better episodic memory relative to placebo. In another study of acute administration of CBD in recreational MJ users who smoked once a month for at least a year, Morgan et al. (2010) reported that those who smoked MJ strains low in CBD performed worse on a verbal memory task than those who smoked MJ strains high in CBD. Similarly, an extension of that study examined THC and CBD levels obtained via hair samples and found that higher levels of CBD were associated with better recognition memory relative to undetectable CBD levels (Morgan et al., 2012). While the majority of studies have investigated the impact of CBD on verbal memory, Borgwardt et al. (2008) utilized a Go/No Go Task, which measures inhibitory function, to examine the functional impact of CBD in healthy volunteers. Functional imaging results revealed that while THC reduced activation in the right inferior frontal and anterior cingulate gyrus, CBD deactivated the left temporal cortex and insula relative to placebo. The authors concluded that THC may attenuate the activity of brain regions that mediate response inhibition, while CBD altered function in regions not typically implicated in response inhibition. Bhattacharyya et al. (2010) also found that intravenous administration of THC and CBD had opposite effects on brain activation patterns across a number of brain regions during the completion of memory, inhibitory, visual, and affective measures. Taken together, these results further suggest that THC and CBD appear to affect the brain very differently, with CBD demonstrating potential for mitigating adverse cognitive consequences that have been widely observed in recreational MJ smokers. The current study included individuals using products high in CBD as well as those using products high in THC; future analyses which include quantified levels of THC, CBD and several other cannabinoids from participants' actual MMJ products are planned in order to examine the specific relationship between MJ constituents and cognitive performance and to determine if some cannabinoids prove beneficial to cognitive status.

In addition, it may be that some of the adverse consequences noted in recreational MJ users were not noted in the current study as all enrolled patients are considered beyond the age of neurodevelopmental vulnerability. Longitudinal studies have shown that the brain continues to develop throughout the late twenties (Giedd et al., 1999); however, all participants included in the current analysis were over 30 years of age. Numerous research studies have found that recreational MJ users with teenage onset experience the most pronounced cognitive deficits (Lisdahl et al., 2013,2014; Jacobus and Tapert, 2014), and those with later onset of use often perform more similarly to non-MJ smoking healthy control subjects (Gruber et al., 2012a; Sagar et al., 2015). Accordingly, our adult MJ onset sample may not be as vulnerable to the negative impact of MJ, specifically products high in THC, as those who use MJ during periods of developmental vulnerability.

### Limitations and future directions

Although data from the current investigation have generated interesting preliminary findings, it is important to consider these results in light of several limitations. As a pilot study, we have thus far only investigated the impact of MMJ use on measures of executive functioning. Future studies should explore additional cognitive domains. In addition, the sample size is inherently modest in nature. However, additional subjects continue to be recruited and enrolled. With additional subjects and increased statistical power, future analyses can employ regression models in order to determine whether MMJ use patterns, product type, constituent ratios, indications for use, or other additional factors predict cognitive performance, mood, or symptom improvement. For example, while a number of patients smoke or vaporize MJ flower products, several also consistently used tinctures, oil, or edibles. In order to inform policy and guidelines for use, research is needed to determine whether certain modes of use have a differential impact on cognition, quality of life, and clinical and medical symptoms. Similarly, information gathered from laboratory analyses, which provide information about the cannabinoid profiles of each product, may provide critical data regarding the impact of individual constituents and eventually, the impact of specific ratios (i.e., THC: CBD) on cognitive and clinical measures. In the current observational study, patients are free to use products of their choice, and often “test out” a variety of products in order to determine what works best for them, especially in the early stages of treatment, which likely increases ecological validity. In addition, with only a handful of dispensaries available to those within the Greater Boston Area, patients in the current study have reported being limited to what products are currently available, and are sometimes forced to change products due to changes in availability. Without statewide or federal regulations to maintain quality control, products may also vary in potency and composition over time or across dispensaries. With larger sample sizes, we aim to explore these questions more fully. For example, we plan to conduct analyses which examine the possibility that high CBD-containing products may have contributed to the positive changes in clinical outcomes and perhaps mitigated some of the adverse cognitive consequences that have historically been observed in recreational MJ users.

Further, while this study employed a pre-post treatment design to assess within-subject changes, given its observational nature, a group of individuals taking placebo or receiving a “sham” treatment could not be utilized; participants in this investigation acquire MMJ products at their own discretion from local dispensaries and/or caregivers, and therefore a placebo arm is not possible. In subsequent work, we plan to utilize clinical trials with more robust double-blind procedures; however, thus far the Schedule I classification of MJ has impeded efforts to conduct these types of clinical trials.

As the current study design requires participants to complete repeated administrations of neurocognitive assessment, we cannot exclude the possibility of practice effects impacting study findings despite attempts to minimize this potential confound. While tasks completed at the baseline visit would be *familiar* to subjects at Visit 2, it is unlikely that practice effects would persist following a 3-month interval between study visits. For example, a study of practice effects in serial neuropsychological testing found no effects on the Letter Number Sequencing and Trail Making tasks even with weekly administration (Beglinger et al., 2005)—a much more frequent schedule of testing than utilized in the current study. Studies noting practice effects on the Stroop test have typically utilized a daily to weekly administration, also a significantly more frequent interval than 3 months (Gul and Humphreys, 2015). In addition, we utilized alternate versions of test measures at Visit 2, further reducing the risk of practice effects. For complex tasks like the WCST, where no alternate version exists, a computerized version of the task, which terminates upon the completion of a specific number of correct categories without any clarification of the rules, was used in order to reduce potential task learning.

In the past, we have had success in studies of recreational users using TLFB procedures to track MJ use (Gruber et al., 2012a,b, 2014; Sagar et al., 2015, 2016; Dahlgren et al., 2016). While MMJ patients appear to be truthful and accurate in providing data on frequency and magnitude of use, the accuracy of their self-reports cannot be confirmed. However, to address this challenge, reports of use during phone check-ins were verified and reviewed at patients' second in-person visit. In general, quantification of MJ use continues to be a challenge in all research studies, as unlike other substances, no standard measure of use is available. This is especially true when considering concentrated products, which often contain high levels of THC (i.e., greater than 60%) in a very small volume or weight. Although MMJ use information was collected and primary MMJ use variables are reported (i.e., days used per week, times used per day), future studies will explore the impact of MMJ use patterns (frequency, mode, constituent ratios, age of initiation, duration of use, mode of use, etc.) and MMJ product composition (i.e., THC vs. CBD), analyses which are planned once larger sample sizes are achieved. Clinical trials geared specifically toward assessing individual MMJ products are still needed to help address issues of confounding variables and to provide additional data regarding the myriad of questions, which only continue to grow as we learn more about MMJ.

Additionally, it will also be important to ascertain whether the improvements noted in the current study persist over longer periods of time. For this reason, the current study is designed to continue to track subjects' cognitive performance and clinical state over the course of 1 year of treatment. Future studies should also investigate whether specific clinical indications for MMJ use impact study results. While some studies have already begun to investigate the effects of cannabinoid-based treatments for specific illnesses or symptoms, such as pain (Eisenberg et al., 2014; Baron, 2015), multiple sclerosis (Flachenecker et al., 2014; Patti et al., 2016), and epilepsy (Press et al., 2015; Devinsky et al., 2016), it is critical to assess whether use of MMJ and related products differentially impact cognition within these populations. Finally, future investigations should aim to explore the mechanism of the improvement noted in the current study. For example, as results primarily indicate improvements in task completion times, more focused examinations of the effects of processing speed may be warranted.

Although our research has begun to address the potential impact of MMJ treatment on cognition, more questions than answers remain. Our findings underscore the need for additional, expanded investigation and exploration. With the nation in the midst of a “green rush” (Silver, 2016) it is imperative to clarify issues including the ways in which recreational and medical MJ use differ. Further, given previous studies assessing the impact of recreational MJ use in adolescents or emerging adults (Crean et al., 2011; Gruber et al., 2012a,b, 2014; Jacobus and Tapert, 2014; Lisdahl et al., 2014; Sagar et al., 2015, 2016; Dahlgren et al., 2016), it will be critical to determine if the same decrements are noted in adult consumers who use MMJ for short or long-term symptom alleviation. Questions regarding how MMJ use impacts quality of life, sleep, clinical state and other important measures remain and are critical areas for further longitudinal investigations.

Given the fact that more than 22 million Americans report current use of recreational MJ (Center for Behavioral Health Statistics and Quality, 2015) and more than 1 million are certified for MMJ use (Procon.org^4^), it is in the public's best interest to develop a robust, evidence-based understanding of both the positive and negative effects of MMJ use on various aspects of functioning: cognition, quality of life, physical and emotional health. Despite attempts to conduct empirically sound clinical research, a number of barriers remain. Restrictions on MJ research and many MJ-related products, including those with little to no THC, stem exclusively from MJ's status as a Schedule I substance. Budding efforts have emerged to move the field forward, including both the introduction of the CARERS Act (Compassionate, Access, Research Expansion, and Respect States Act, 2015^5^) and the Therapeutic Hemp Medical Access Act (Therapeutic Hemp Medical Access Act, 2015). Both of these acts seek, among other agenda items, to exclude CBD as well as low THC/high CBD strains of MJ (in other words, non-psychoactive products) from the current definition of “marijuana” under the CSA in order to facilitate access to MMJ patients. Although both bills were introduced over a year ago, their status is still pending. As it currently stands, non-psychoactive (low to no THC) industrial hemp-derived products, which are widely available for sale, cannot be studied through clinical research trials given current restrictions. Accordingly, consumers can gain access to these products that cannot be assessed by clinical researchers with regard to efficacy and safety. In spite of these obstacles, some progress has recently provided hope to those who seek alternative treatments. As of April 2016, the DEA approved the first study of smoked MMJ for a clinical trial of Post-Traumatic Stress disorder (Clinicaltrials.gov^6^). Although the DEA announced earlier this year that it was reconsidering the classification of MJ, a decision released in August 2016 revealed that MJ will remain a Schedule I substance (Department of Justice, 2016a). In an effort to promote clinical research studies of MJ, the DEA instead indicated that it will allow “entities” to apply for a DEA registration to grow their own MJ for research (Department of Justice, 2016b); this stands in contrast to the current protocol mandating that all whole plant-derived MJ products used for clinical trials be sourced from the National Institutes of Drug Abuse (NIDA). While investigations of the purified extracts of individual cannabinoids are currently being studied, most notably the GW Pharma trials of Epidiolex, a purified CBD product for children with intractable seizure disorders, some have reported that products derived from whole plant botanicals provide greater symptom amelioration and relief (Grinspoon and Bakalar, 1997; Joy et al., 1999), underscoring the need for additional sources of MJ products for clinical trials.

## Conclusions

Data from the current investigation provide preliminary evidence that after 3 months of treatment, MMJ users did not experience executive functioning deficits, which are often observed in regular, recreational MJ users. In fact, MMJ patients evidenced improvement in certain aspects of performance on these measures, particularly with regard to time required to complete tasks. Further, patients reported some improvements on measures of clinical state and general health as well as a decrease in conventional pharmaceuticals, notably opiate use, which was reduced by 42% between the baseline and Visit 2 assessment. While future studies are needed to further examine the impact of MMJ, research is impeded by a number of federal and state restrictions. It is imperative, however, that sound research, including well-controlled clinical trials of MMJ products, many of which are already widely used by patients, are thoroughly examined. As the “green rush” pushes forward, gaining momentum as states continue to adopt less restrictive policies, we cannot afford for research to continue to lag behind.

## Author contributions

SG conceptualized and designed the current study in consultation with SL. KS and MD assisted SG in manuscript preparation. SG, KS, MR, and RS recruited patients, carried out study procedures, and administered neuropsychological and clinical assessments. MD also completed statistical analyses.

## Funding

This project was funded by generous private donations to the Marijuana Investigations for Neuroscientific Discovery (MIND) Program at McLean Hospital.

### Conflict of interest statement

The authors declare that the research was conducted in the absence of any commercial or financial relationships that could be construed as a potential conflict of interest.

## Footnotes

^1^Medical Marijuana ProCon.org. (2016). Retrieved June 16, 2016, from http://medicalmarijuana.procon.org/ Procon org (2016). Number of Legal Medical Marijuana Patients. Retrieved from http://medicalmarijuana.procon.org. March 3).

^2^Retrieved June 16, 2016, Available online at: http://www.dea.gov/index.shtml

^3^Comprehensive Drug Abuse Prevention and Control Act of 1970, Pub. L. No. 91-513, 84 Stat. 1236, 21 U.S.C. §812.

^4^Medical Marijuana ProCon.org. (2016). *Retrieved June 16, 2016, from* http://medicalmarijuana.procon.org/

^5^Compassionate Access, Research Expansion, and Respect States Act of 2015, S. 683, 114th Cong. (2015).

^6^Clinicaltrials.gov. (2016). Retrieved August 29, 2016, from https://clinicaltrials.gov/ct2/show/NCT02759185?term=%22sisley%22&rank=1
